# Intra-partum fever and cerebral palsy in Khartoum, Sudan

**DOI:** 10.1186/1756-0500-6-163

**Published:** 2013-04-24

**Authors:** Hala Abdullahi, Mohamed Satti, Duria A Rayis, Abdulmutalab M Imam, Ishag Adam

**Affiliations:** 1 Department of Obstetrics and Gynecology, University of Khartoum, Khartoum, Sudan

**Keywords:** Cerebral palsy, Labor, Pregnancy, Sudan

## Abstract

**Background:**

Cerebral palsy (CP) is a major cause of childhood disability. There are various maternal and neonatal predictors associated with the development of CP, and they are variable across different populations. This case–control study was designed to investigate maternal and neonatal predictors of CP at Khartoum pediatric neurology clinics. Data (maternal sociodemographic characteristics and neonatal expected predictors) were collected from mothers of children with CP and healthy controls using questionnaires.

**Results:**

One hundred and eleven cases of CP and 222 controls were included. Spastic CP was the most common type (69.4%). In logistic regression, maternal age, parity, birth weight, and sex were not associated with CP. However, maternal fever (OR = 8.4, CI = 2.3–30.5; P = 0.001), previous neonatal death (OR = 5.4, CI = 1.8–16.2; P = 0.003), and poor sucking (OR = 30.5, CI = 10.0–93.1; P < 0.001) were predictors of CP.

**Conclusions:**

Fever during labor is a significant risk factor for developing CP in children. Further efforts are required for labor management to prevent CP in this setting.

## Background

Cerebral palsy (CP) is a group of permanent disorders of development of movement and posture, causing activity limitation, and it is attributed to non-progressive disturbances that occur in the developing fetal or infant brain [1]. CP is the most common physical disability in children, affecting 1–2 per 1000 live births [2-5].

The exact etiology of CP is not yet well understood, and brain lesions are thought to be associated with perinatal events of varying causes. Several predictors for the development of CP have been identified in various settings, such as maternal age, pre-eclampsia, chorioamnionitis, small for gestational age, multiple births, assisted reproduction, a relative with CP, breech position, bleeding at any time in pregnancy, male sex, and multiple miscarriages [4,6-15]. These factors are variable among different populations.

The predictors of CP need to be determined to generate basic data for producing interventional measures to reduce the risk of CP. While there are a lot of published data on the epidemiology of CP in other countries, there are no published studies regarding the risk factors for CP in Sudan. In this case–control study, we aimed to investigate the predictors of CP in Khartoum to add to previous knowledge on neonatal morbidity and mortality in this country [16,17].

## Methods

A case–control study was conducted at the pediatric neurology clinic in Khartoum, Sudan, between January and June 2012. A sample size of 110 subjects was calculated based on a two-sided hypothesis test using Epi Info with 80% power to detect a difference of 5% at α=0.05 and a confidence interval of 95%, and 10% of non-responders were expected. The mothers of children who had already been diagnosed with CP (based on the medical history, a clinical examination, and imaging of the brain) and who attended follow-up were interviewed using a pretested questionnaire. Cases with CP were classified into three major categories; spastic, dyskinetic, and ataxic subtypes.

Two controls for each case were selected from the hospital, matched for time and place of birth as much as possible after exclusion of major congenital anomalies. After obtaining informed consent, pretested questionnaires were applied for each mother/guardian to gather maternal sociodemographic characteristics, and information on labor and perinatal and neonatal events that were expected to be predictors for CP (age, parity, antepartum hemorrhage, intra-partum fever, mode of delivery, gestational age, birth weight, and admission to the nursery).

### Statistical analysis

Data were analyzed using SPSS (Social Package for Scientific Statistic) version 17.0. Means and proportions were compared between the cases and controls using the *t*-test and *χ*^2^ test as appropriate. Univariate and multivariate analyses were performed with CP as the dependent variable, and maternal and neonatal factors as independent variables. P < 0.05 was considered statistically significant.

### Ethics

Ethical approval was obtained from the Sudan Medical Specialization Board.

## Results

There were 111 cases of CP and 222 controls. Different types of CP were observed as follows: spastic (77; 69.4%), dyskinetic; (24; 21.6%), ataxic (seven; 6.3%), and mixed (three; 2.7%). The age ranged from 1–11 years, with a mean (SD) of 4.1 (2.9) years. The mean maternal age (31.5 [7.5] vs. 32.5 [6.8] years, P = 0.256) and paternal age (40.8 [9.2] vs. 42.0 [8.9] years, P = 0.263) were not significantly different between the CP group and controls. The mean birth weight was slightly lower (not significant) in the CP group compared with controls (2970.2 [644.4] vs. 3019.0 [651.5] g, P = 0.534).

While there was no significant difference in most of the sociodemographic characteristics between the CP group and controls, significantly more mothers in the CP group had a history of neonatal death (16 [14.4%] vs. 9 [4.1%], P = 0.001), had previous newborns with CP (7 [6.3%] vs. 1 [0.5], P = 0.002), and had fever during delivery of the index baby (22 [19.8%] vs. 4 [1.8%], P < 0.001) compared with the controls. Significantly more children in the CP group presented in breech (15 [13.5%] vs 14 [6.3%] P = 0.03], had poor sucking (53 [47.7%] vs 6 [2.7%], P < 0.001), and were admitted to the neonatal care unit earlier in their life (23 [20.7%] vs 7 [3.2%], P < 0.001) (Table 1).

**Table 1:** Comparison of sociodemographic characteristics of CP cases and controls

**Variables**	**Cases with cerebral palsy (n= 111)**	**Healthy controls (n=222)**	**P**
*Previous maternal history of*			
Miscarriage	23(33.0)	43(19.4)	0.772
Preterm delivery	3(2.7)	4(1.8)	0.690
Neonatal death	16(14.4)	9(4.1)	0.001
Baby with cerebral palsy	7(6.3)	1(0.5)	0.002
Cesarean delivery	7(6.3)	22(9.9)	0.309
*Variables related to pregnancy being questioned*			
Assisted conception	3(2.7)	6(2.7)	1.000
Antepartum hemorrhage	7(6.3)	6(2.7)	0.135
Multiple conception	6(5.4)	8(3.6)	0.563
Male gender	59(53.2)	131(59.0)	0.348
Induction of labor	5(4.5)	11(5.0)	1.000
Fever during labor	22(19.8)	4(1.8)	<0.001
Vaginal delivery	99(89.2)	183(82.4)	0.824
Cesarean delivery	12(10.8)	39(17.6)	0.824
Breech presentation	15(13.5)	14(6.3)	0.03
Admission to the neonatal care unit	23(20.7)	7(3.2)	<0.001
Poor sucking	53(47.7)	6(2.7)	<0.001

Parity, a history of miscarriage, antepartum hemorrhage, gestational age, induction of labor, and mode of delivery were not significantly associated with CP. Breech presentation (OR = 2.3, CI = 2.1–5.0; P = 0.031) and a previous child with CP (OR = 14.9, CI = 1.8–122.4; P = 0.012) were significant risk factors in univariate analysis but not in multivariate analysis (Table 2).

**Table 2:** Univariate and multivariate analyses for possible predictors of cerebral palsy among children in Khartoum, Sudan

**Variables**	**Univariate analysis**			**Multivariate analysis**		
	**OR**	**95% CI**	**P**	**OR**	**95% CI**	**P**
Maternal age	1.0	1.0—1.1	0.255	1.0	0.9—1.0	0.517
Parity	1.1	1.0—1.2	0.093	1.1	0.9—1.4	0.211
History of miscarriage	1.1	0.6—1.9	0.771	1.0	0.4—2.3	0.991
Antepartum hemorrhage	2.4	0.8—7.4	0.12	1.9	0.4—8.4	0.397
Previous baby with cerebral Palsy	14.9	1.8—122.4	0.012	8.2	0.7—92.4	0.088
Previous neonatal death	4.0	1.7—9.3	0.001	5.4	1.8—16.2	0.003*
Previous cesarean delivery	0.6	0.3—1.5	0.276	0.2	0.04—1.0	0.058
Male gender	0.8	0.5—1.2	0.303	0.8	0.4—1.5	0.441
Induction of labor	0.9	0.3—2.7	0.856	1.0	0.2—4.4	0.987
Fever during labor	31.5	4.5—40.2	<0.001	8.4	2.3—30.5	0.001*
Cesarean delivery	1.1	0.8—1.5	0.524	0.8	0.5—1.4	0.485
Breech presentation	2.3	1.1—5.0	0.031	0.6	0.2—1.9	0.374
Birth weight	1.1	0.8—1.6	0.535	0.9	0.6—1.4	0.660
Gestational age	1.1	1.0—1.2	0.08	0.9	0.8—1.1	0.531
Admission to the neonatal unit	8.0	3.3—19.4	<0.001	1.2	0.3—5.4	0.793
Poor sucking	32.9	13.5—80.3	<0.001	30.5	10.0—93.1	<0.001*

*Adjusted for confounders.

In multivariate analysis, maternal fever (OR = 8.4, CI = 2.3–30.5; P = 0.001), previous neonatal death (OR = 5.4, CI = 1.8–16.2; P = 0.003), and poor sucking (OR = 30.5, CI = 10.0–93.1; P < 0.001) were predictors for CP (Table 2).

## Discussion

The main findings of this study were that maternal fever, previous neonatal death, and poor sucking were significant predictors of CP in Khartoum, whereas maternal age, parity, birth weight, and sex were not associated with CP. In this study, only 70% of these children had spastic CP. Previous studies in other developing countries, such as India [18] and Egypt [5], showed that spasticity is the most common type of CP. Paternal and maternal age have been reported to be associated with an increasing risk of CP in other studies [12,19].

Gestational age and birth weight were not associated with CP in the current study. More than half of all children with CP are not preterm deliveries [20-22]. However, prematurity and low birth weight have been found to be associated with CP in other populations [4,12]. Recall and selection bias might be responsible for masking the true effects of birth weight and gestational age in the current study. Generally, the actual birth weight and gestational age are only reliable if a prospective or longitudinal study is conducted, which is difficult in a setting such as Khartoum.

A history of having a child with CP was associated with CP in univariate but not in multivariate analysis in our study. Previous poor obstetric history, such as miscarriage, intrauterine fetal death, neonatal death, or a previous child with disability, is associated with an increasing risk of CP [23,24]. Having a child with CP appears to increase the risk in subsequent children, possibly because of genetic predisposition [25-27]. Early feeding difficulties manifesting as poor sucking abilities was a significant predictor for the development of CP in our study, as well as in other studies [28,29].

In the current study, mothers with a history of fever during labor had a nine times greater risk of having newborns with CP. This may reflect suboptimal antenatal care or suboptimal management of labor. We have recently observed that obstructed labor constitutes a major threat to the mother and fetus in Eastern Sudan [30]. Furthermore, we have recently found that infection is the leading cause for admission to the nursery for newborns born in hospital [17]. Unfortunately, even in the capital, Khartoum, suboptimal care of labor and its management are responsible for high perinatal death, which is 51 per 1000 births [31]. Therefore, the current study supports several previous studies showing that maternal infection at the time of delivery is an important risk factor for CP [4,7,8,10,14,15].

One of the limitations of this study is that the delivery data were collected from mothers many years after the event. Therefore, inherent risks of recall bias can be expected. This is especially relevant when women were asked about fever in labor, which can be a subjective symptom, and we did not refer to the delivery notes. However, this question was explained as a high temperature being confirmed by the attending treating doctor or requiring intervention to treat it. The study group was a sample from pediatric neurology clinics over a 6-month period. This may have skewed the sample of children with CP because they do not represent all patients in the community. Less severe cases may not have been well represented and not all adhered to follow-up because of various barriers, including limited transport and financial resources. Using hospital-based controls is convenient for data collection, but they are not ideal because they may have other confounders that made them present to the hospital.

## Conclusions

Mothers having previous newborns with CP, poor sucking, and fever during labor are predictors for developing CP in children. There should be further efforts antenatally and during labor to introduce measures and management options for reducing CP in this setting.

## Competing interests

The authors declare that they have no competing interests.

## Authors’ contributions

HA, MS, and IA designed the study. DAR and AI conducted the clinical part of the study. DAR and IA performed statistical analysis. All the authors read and approved the final version.
